# Role of *MS1* homolog *Ntms1* gene of tobacco infertility

**DOI:** 10.1515/biol-2021-0087

**Published:** 2021-08-24

**Authors:** Qian Chen, Tingting Zhao, Lili Duan, Zejun Mo, Maozhu Tian, Zhenhua Li, Yang Liu, Renxiang Liu

**Affiliations:** Guizhou University College of Agriculture/College of Tobacco, Guizhou Provincial Key Laboratory of Tobacco Quality Research, Guiyang 550025, China

**Keywords:** *MS1* gene, tobacco, fertility, homologous cloning

## Abstract

The sterile line is the basis of crop heterosis utilization. To broaden the sources of male sterility in tobacco, the *Ntms1 (Nicotiana tabacum* L. *ms1)* gene was cloned from the tobacco variety K326 by homologous cloning based on the *Cams1* (*Capsicum annuum* L. *ms1*) gene sequence of male-sterility genes in pepper. The protein structure and physicochemical properties of the two genes were determined by bioinformatics analysis, and the function of the *Ntms1* gene was verified by the CRISPR/Cas9 system. The results showed that the sequences of Ntms1 and Cams1 were 85.25% similar, and plant homeodomains were found in both genes; the physical and chemical properties were also very similar. It is speculated that the *Ntms1* gene had the same function as the *Cams1* gene in controlling male sterility. Compared to the wild-type plants, the filaments of the *Ntms1* knockout mutant plants were shorter, and the stamen was shorter than the pistil. The anthers did not develop fully and had few viable pollen grains; the tapetum and the anther wall had developed abnormally, and the anther chamber was severely squeezed. The malondialdehyde content in the mutant plants was significantly higher than that in the wild-type plants, while self-fertility was significantly lower in the mutant plants. The results showed that the *Ntms1* gene plays an important role in regulating fertility in tobacco.

## 1 Introduction

Male sterility is an important agronomic trait in studies of crop heterosis utilization. It is a genetic phenomenon defined by the inability of plants to produce normal anthers, pollens, or male gametes during sexual reproduction, while the pistil functions normally [1]. The sterile males are used to produce hybrids that can save artificial castration, reduce seed production costs, and improve seed purity. To date, 13 genes associated with male sterility have been cloned from Arabidopsis and maize. Among them, the *MS1* gene, which has been proven to be a key gene regulating pollen formation, is mainly expressed when microspores are released from the tetrad and encodes a plant homeodomain (PHD)-containing nuclear protein [2]. PHD is closely related to the composition of the pollen wall and the tapetum development and does not affect other floral organs. They cause pollen abortion, and thus, can be used in production [3]. The *PTC1* gene in rice was found to be homologous to the *MS1* gene in Arabidopsis, which also encodes a PHD domain and shows similar functions in regulating the development of villus cells and the pollen wall [4]. The homologous *Zmms1* gene in maize was cloned from the *Osms1* gene in rice by homologous cloning, and the results of the bioinformatics analysis showed that both *Zmms1* and *Osms1* were very similar to the members of the PHD domain family. It is speculated that the *Zmms1* gene, along with *Osms1*, may control fertility [5]. The CRISPR/Cas9 system was used to develop male-sterile lines without transgenic maize and obtain stable genetic lines [6]. Using the *MS1* gene in *Arabidopsis thaliana* as a reference sequence, bioinformatic methods were applied to predict and analyze the amino acid sequences encoded by Brassica crops such as rape, Chinese cabbage, and cabbage. The results showed that the *MS1* gene in Brassica crops belonged to the PHD finger family, and its highly conserved sequence was involved in the regulation of pollen development and maturation [7]. Using the CRISPR/Cas9 system to create targeted mutations in *MS1*, a double displacement code mutation was introduced into *MS1*, which resulted in complete male sterility in wheat varieties Fielder and Gladius [8]. Three homologous sequences of the *MS1* gene modified by the CRISPR/Cas9 system could rapidly generate male-sterile wheat lines [9]. The CRISPR/Cas9 system was used to mediate the knockout of the *MS1* gene, causing extremely low expression levels of this gene in the anther, which had missing spikelets; the self-fertilization rate was also significantly reduced [10]. The *Cams1* gene in pepper is highly homologous to the *MS1* gene in *Arabidopsis thaliana*. It contains the PHD domain and two low complexity domains, which control pollen fertility. When compared with fertile plants, the number of pollens in sterile pepper plants was significantly reduced [11]. In summary, the role of the *MS1* gene in the regulation of fertility has been confirmed in major crops such as rice, maize, rape, wheat, and pepper. However, it has not yet been reported whether the *MS1* gene regulates male sterility in tobacco.

The tobacco leaves are the most economically important part of the plant. For controlling the various layouts and areas of tobacco production, in addition to the direct use of sterile hybrids, pure line varieties bred by hybridization are often transformed into sterile lines for tobacco production. The proportion of sterile lines in tobacco production is as high as 80%. However, the sterile lines used in tobacco production are derived only from the cytoplasmic sterile genes of *N. suaveolens*, and there is a single source of sterility in genes; the cytoplasm is easily infected by pathogenic bacteria, which affects the production and application of tobacco hybrids and sterile lines [12,13]. Therefore, broadening the source of male sterility in tobacco is very important for stable tobacco production. While studying male sterility in tobacco, previous studies had mostly focused on cytoplasmic male sterility and found *Atp6*, *Atp9*, *Arf25*, and *Arfb* sterile cytoplasmic genes [14]. Tobacco and pepper belong to the Solanaceae family and have high homology. Therefore, in this study, the *Ntms1* gene was cloned from tobacco by the homologous cloning method based on the *Cams1* genome sequence of pepper, and the knockout vector was constructed by the CRISPR/Cas9 technology. The function of the *Ntms1* gene was identified, and sterile tobacco plants were produced, which have immense scientific significance and practical value for broadening the source of male sterility in tobacco.

## 2 Materials and methods

### 2.1 Tobacco plant varieties

The tobacco variety used in the experiment was the main variety K326, usually used in tobacco production and preserved and provided by the Guizhou Tobacco Quality Key Research Laboratory.

### 2.2 Carriers and strains

*Escherichia coli* DH5α and *Agrobacterium tumefaciens* EHA105 were preserved and provided by the Guizhou Tobacco Quality Research Key Laboratory. The TA cloning vector pMD18-T was provided by the Shaanxi Border Biotechnology Co., Ltd. The target design and comparison of the CRISPR/Cas9 recombinant vector MSG2 were performed in the Guizhou Key Laboratory of Tobacco Quality Research, and the synthesis of the CRISPR/Cas9 recombinant vector was performed by Baige Gene Technology (Jiangsu Co., Ltd).

### 2.3 Total RNA extraction and cDNA synthesis

Total RNA was extracted using the RNAiso Plus Tri Zol (Ta Ka Ra). The cDNA was synthesized using the Poly Attract^®^ mRNA Isolation System III with Magnetic Stand kit from Promega, which was purchased from Bao Bioengineering (Dalian) Co., Ltd. The reverse transcription system and reaction procedure are listed in Table S2.

### 2.4 The creation of mutants

#### 2.4.1 Cloning and vector construction of target genes

The amino acid sequence Cams1 (LOC107852993) of the MS1 protein in pepper was obtained from the NCBI website (https://www.ncbi.nlm.nih.gov/). The amino acid sequence was compared to that in common tobacco to find the protein that had the highest homology with MS1 (GenBank accession number: LOC107813657). The query coverage of the two sequences reached 100% (Figure S10). The specific primer 657 – F (ATGGTGGTAA TGAATGGAAGGCC)/657 – R (TCAGGCAGCA TTAGTCAAGC) was designed with this sequence using the Primer 5.0 software, cloned according to the method developed by Sun et al. [15], and sequenced in the Shaanxi Brad Biotechnology Co., Ltd.; the sequence was called *Ntms1*. The *Ntms1* gene (the PHD finger protein) was constructed by the CRISPR/Cas9 vector. Initially, the target genes were sequenced to eliminate SNP interference, thus ensuring highly accurate targeted editing, and then gRNA was constructed according to the methods developed by Okada et al. [8] (See Appendix for the construction process and procedures of CRISPR/Cas9 vector).

#### 2.4.2 Bioinformatics analysis

The ORF finder (https://www.ncbi.nlm.nih.gov/orffinder/) in NCBI was used to identify the open reading frames of the *Cams1* gene in pepper and the *Ntms1* gene in tobacco. The ProtParam system (https://web.expasy.org/protparam/) in the ExPASy database was used to analyze the physical and chemical properties of the isoelectric point (PI) and the extinction and instability coefficients of proteins. The ProtScale online software (https://web.expasy.org/protscale/) was used to estimate the hydrophilicity of the proteins. The InterPro online software (http://www.ebi.ac.uk/interpro/) was used for analyzing the protein domain. The NetPhos 3.1 online website (http://www.cbs.dtu.dk/services/NetPhos/) was used to analyze the protein phosphorylation sites. The SOPMA and Phyre2 were used online to analyze and predict the secondary and tertiary structures of the proteins, respectively. The TMHMM online software (http://www.cbs.dtu.dk/services/TMHMM/) was used to analyze the transmembrane domains in proteins. The Signal P4.1 online software was used to determine whether the proteins contained signal peptides. The DNAMAN software was used to perform multiple alignments of protein sequences and observe the similarity between them. The MEGA7.0 software was used to construct a molecular evolutionary phylogenetic tree for the species selected from the NCBI database, and the phylogenetic relationship between species was observed using the evolutionary tree.

#### 2.4.3 Genetic transformation and screening for positive strains

The genetic transformation technique was applied based on the CRISPR/Cas9 gene-editing technology established by Tian [16]. The identification of positive mutants was based on the screening method developed by Chen et al. [17].

#### 2.4.4 Gene expression analysis of the *Ntms1* gene in mutant plants

The RT-PCR primers PHD_F (ATGTTGGCTTGTGATGTCTG)/PHD_R (TTCCCGCTTGTATTTGTAGTC) were designed based on the *Ntms1* sequence, and the expression of the *Ntms1* gene was analyzed using the tobacco Actin internal reference gene primers, Actin-F (CATGAAGATTAAAGGCGGAGTG)/Actin-R (AACAGTTTGGTTGGAGTTCTGG).

### 2.5 Analysis of the fertility of the mutants

#### 2.5.1 Observation of the floral organ in mutant strains

Wild-type tobacco plants were considered as control. The upcoming opening buds were taken; the anther fullness, stigma, and filament length were observed, and photographs were taken.

#### 2.5.2 Identification of mutant strains associated with drug development

The flower buds of the mutant strains were fixed in a formalin-acetic acid-alcohol solution, and the drug delivery sections were observed according to the method developed by Wang et al. [18].

#### 2.5.3 Identification of pollen grains in mutant plants

At the full bloom stage, plants were cut using a dissecting needle to remove the anther wall before it cracked. All the pollens were picked up after dyeing and mixed in a 200 µL I-KI staining solution. The pollen morphology was observed under the microscope (XHC-L2, 40×) after dyeing. Five fields were randomly selected for each plant, and the number of dyed pollen grains was calculated. The active pollens were the fully developed jacinth, while the inactive and less active pollens were buff.

#### 2.5.4 Detection of the MDA content in mutant strains

The mature leaves from the first (bottom) to the 14th leaf (top) in each mutant were transferred to the laboratory in a wet gauze and measured by the methods developed by Zhang et al. [19].

#### 2.5.5 The estimation of self-fertility rate in mutant strains

After the central flower in the mutant plants opened, ten flowers with slightly red corollas that had been bagged and self-pollinated were randomly selected from each mutant; the seeds were harvested at maturity, and the fruit rate was recorded. The number of seeds per fruit was then calculated, and the relative seed setting rate of the mutant plants was estimated.

### 2.6 Statistical analysis

The Excel 2013 (Microsoft, USA), DPS 14.10 (Hangzhou Ruifeng Information Technology Co., Ltd., China), and Origin (OriginLab, the US) software were used for statistical data analysis. All the data used were the average of three biological replicates; the error bar represents standard deviation (±SD). A “*” sign represents *P* < 0.05, with significant difference; a “**” sign represents *P* < 0.01, with highly significant difference.

## 3 Results

### 3.1 The function of the *Ntms1* gene in controlling fertility

The *Ntms1* gene, which was obtained by homologous cloning of the *Cams1* gene, controls fertility. The results of the bioinformatics analysis showed that the start codon of *Ntms1* is located at the 36th nucleotide, and the stop codon is located at the 2,174th nucleotide, which encodes a total of 712 amino acids. The number of exons is 2,137 bp, and the number of introns is 817 bp. The start codon of *Cams1* is located at the first nucleotide, and the stop codon is located at the 2,100th nucleotide. It encodes a total of 699 amino acids; the number of exons is 2,111 bp, and the number of introns is 584 bp. The coding regions of the two genes overlap, and the difference in the number of encoded amino acids is very small, indicating that the proteins encoded by the two genes are not significantly different (Figure S1). The similarity between Ntms1 and Cams1 proteins was 85.25%. Both were non-secretory and non-transmembrane proteins, and their receptors had protein kinase activity (Figures S2–S6). The hydrophilicity indices of these two proteins were −0.238 and −0.299, respectively, which made them hydrophilic proteins. Additionally, the physical and chemical properties such as molecular weight, isoelectric point, and the number of positively and negatively charged amino acids were very similar (Table 1), and they all had the same PHD domain (Figure S5). The spatial similarity of the secondary structure was also very high (Table S1), and the result of the phylogenetic tree analysis (Figure S8) showed that the homology between the two was 100%. It is speculated that the *Ntms1* gene has the same function as *Cams1* in controlling fertility.

**Table 1:** Physical and chemical properties of Cams1/Ntms1 protein

Protein	Molecular weight (kDa)	Isoelectric point	Negatively charged amino acid (Asp + Glu)	Positively charged amino acid (Arg + Lsy)	Unstable coefficient	Hydrophilic index
Cams1	78.16	8.22	81	87	37.95	−0.238
Ntms1	79.62	8.48	82	91	35.25	−0.299

### 3.2 The effect of the transformant *Ntms1* gene editing

**Figure 1:**
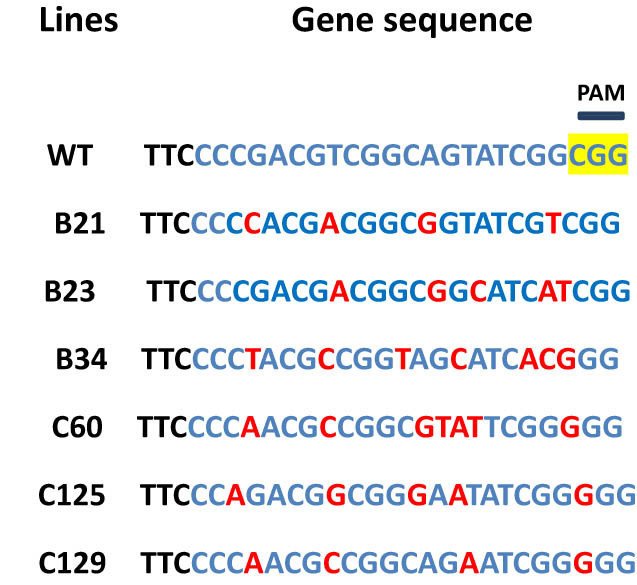
Sequencing results of mutant strains. Blue represents the target site, red represents the mutated base, and yellow represents the PAM site. All of the six strains had multiple base mutations.

**Figure 2:**
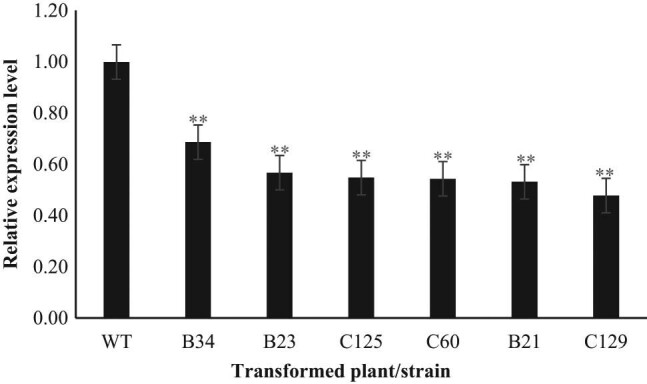
Relative expression of *Ntms1* gene in mutant. Note: All data are the average of three biological repetitions, a “**” sign represents *P* < 0.01, with highly significant difference.

The results showed that the Cas9 gene could be detected by the CRISPR/Cas9 gene-editing technology in six genetically transformed plants (Figure S7), and 4–6 base substitutions occurred at the targeted gene sites of the transformed plants (Figure 1). The expression of the *Ntms1* gene in the six mutant strains was significantly lower (48–69%) than that in the wild-type plants (Figure 2). The results showed that the CRISPR/Cas9 vector played an important role in editing the target gene in the transformed strain, resulting in significant changes in the sequence and expression level of the target gene. The target gene of the transformed strain was mutated.

### 3.3 Significant changes in fertility-related traits of *Ntms1* knockout mutants

#### 3.3.1 Changes in the structure of floral organs in mutant strains

**Figure 3:**
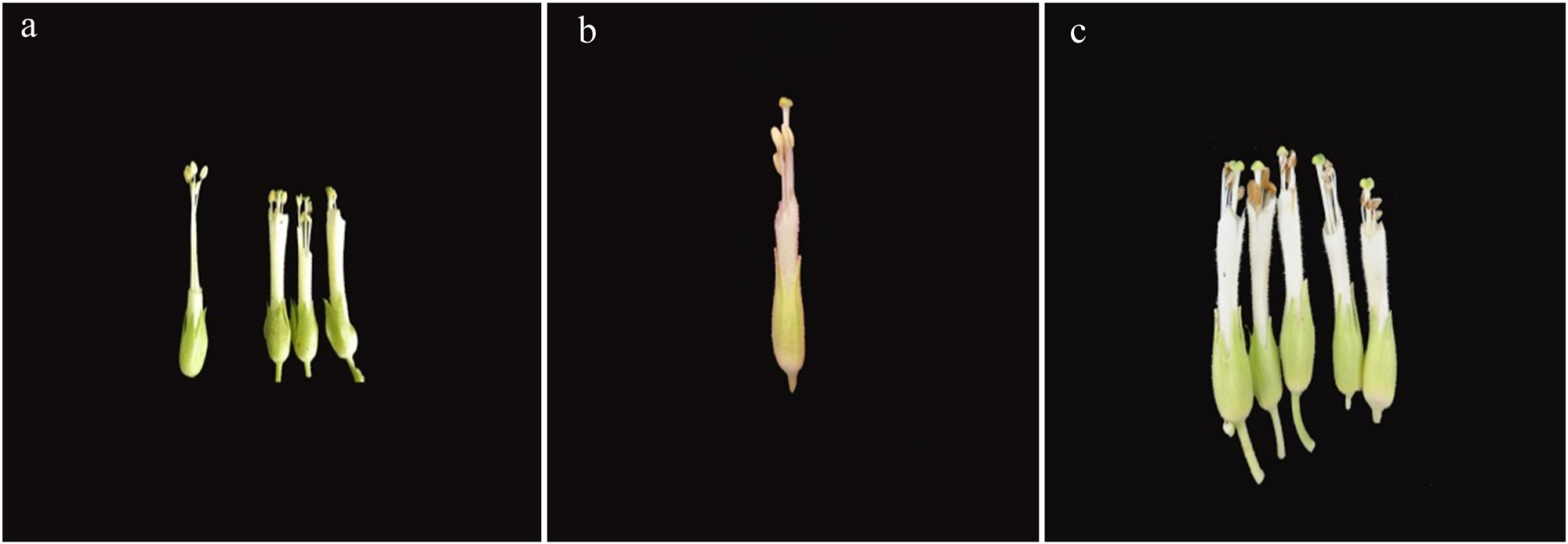
Observation on flower morphology of the mutants and WT. (a) On the left is the wild type, the anther is higher than the stigma. On the right is the mutant, the anther is lower than the stigma. (b and c) Mutant, anther atrophy, growth short and below the stigma.

Observations of the morphology of the floral organ of the mutant and wild-type plants (Figure 3) showed that although both the plants could flower normally, the anther and filament length of the mutant plants changed significantly when compared with those of the wild-type plants. The anthers of the wild-type plants were full and larger than the stigma, and pollen grains were scattered on the surface of the hair. However, the filaments of the mutant were shorter, the anther height was lower than that of the stigma, and self-pollination could not be performed. The anthers were shrunk, and there were few mature pollen grains. The knockout of the *Ntms1* gene led to the shortening of the filament and pollen abortion.

#### 3.3.2 Abnormal development of the anther wall and tapetum in mutant strains

**Figure 4:**
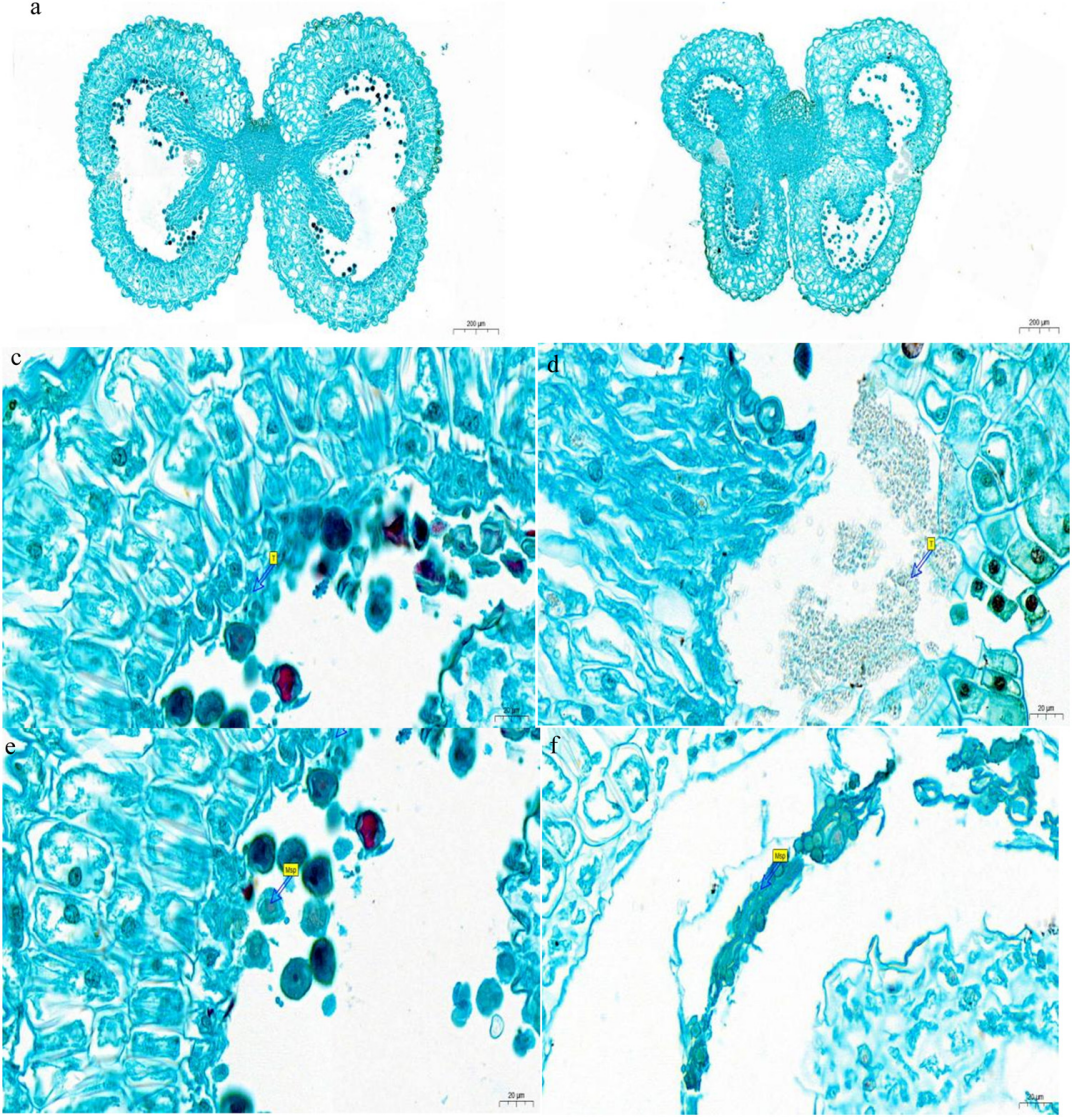
Paraffin section observation of pollen. (a, c, and e) WT. (b, d, and f) Mutant. Msp, microspores. T, tapetum. (a and b), *Bars =* 200 μm. (c–f), *Bars =* 20 μm.

Observations of the anther slices (Figure 4) showed that the edges of the anther wall of the wild-type tobacco were clear, and multiple, nearly spherical pollen grains were evenly distributed in the anther chamber. The anthers in the mutant plants developed abnormally, and the chambers were severely squeezed; death of the tapetum occurred, and the pollen grains were abnormal. This indicates that the deletion of the *Ntms1* gene resulted in the abnormal development of the tapetum, which could not provide the necessary nutrients for microspore development.

#### 3.3.3 Membrane damage in mutants

**Figure 5:**
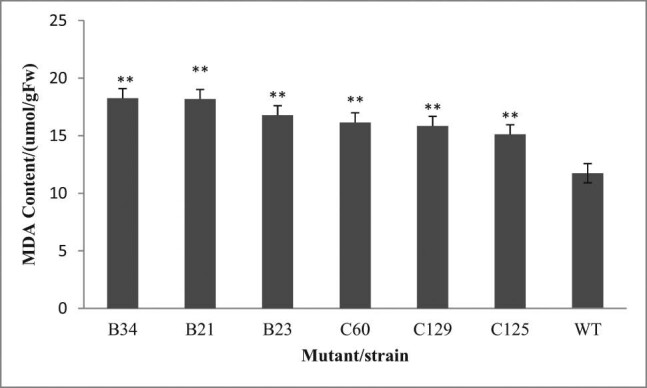
The detection of MDA content. Note: All data are the average of three biological repetitions, a “**” sign represents *P* < 0.01, with highly significant difference.

In a study on male sterility in cotton, a high MDA content caused abnormal metabolism, accumulation and peroxidation of membrane lipids, and pollen abortion [20]. Figure 5 shows that the MDA content of the mutant plants was significantly higher than that of the wild-type plants, indicating that the knockout of the *Ntms1* gene had caused a certain degree of damage to the membrane system, which might have led to infertility.

#### 3.3.4 A significant decrease in the number of viable pollen grains

**Figure 6:**
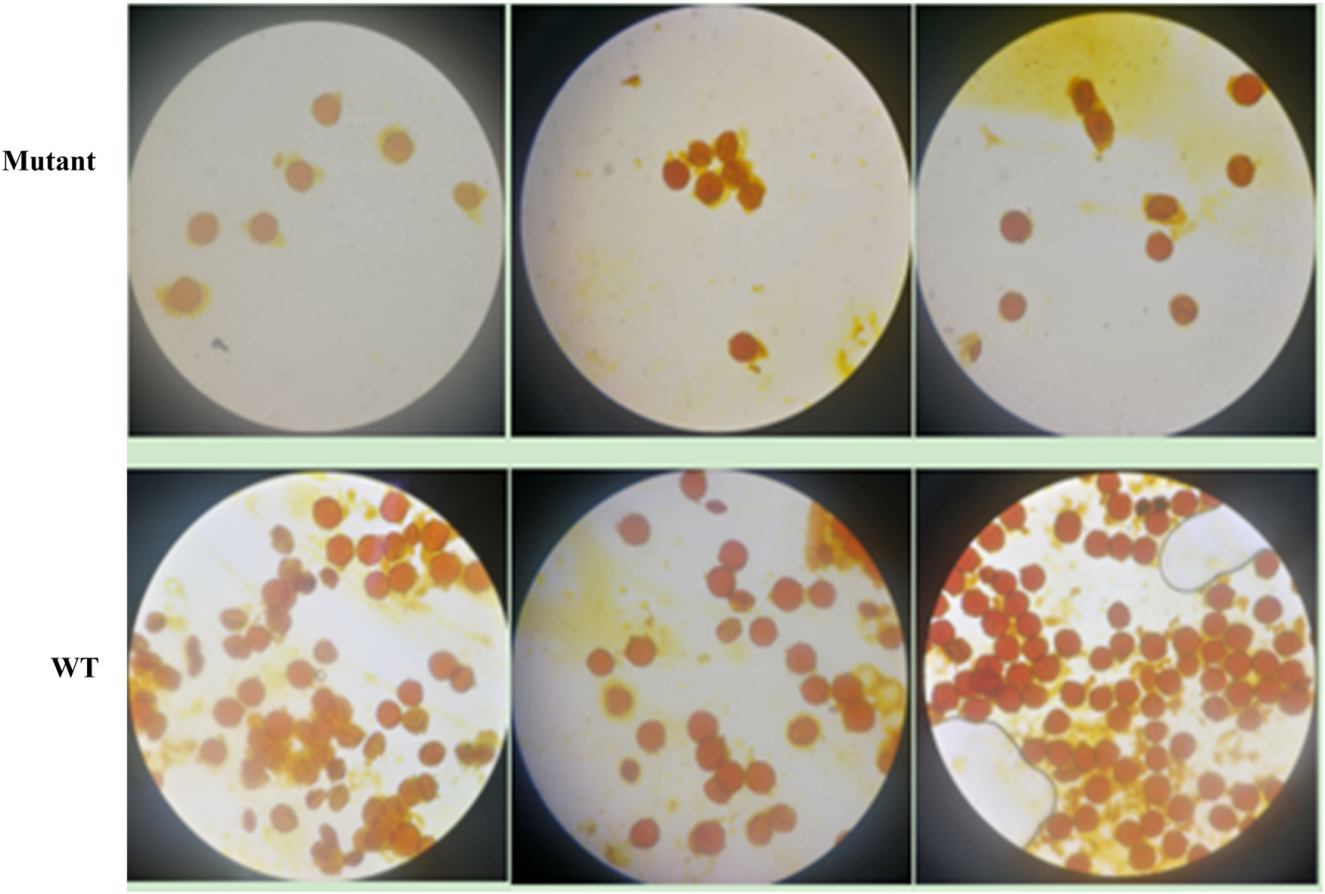
Determination of pollen vigor of mutant and wild-type (upper row: mutant; bottom row: wild type). The number of viable pollen grains in the mutant strain was significantly lower than that of the wild type.

The number of viable pollen grains and pollen viability was the most direct indicators of fertility. The results of the determination of the number of pollen grains and pollen viability in the mutant plants (Table 2, Figure 6) showed that viable pollen grains accounted for 6.4–10.8% of the total pollen grains and were significantly lower than that in the wild-type plants. The color of the viable pollen grains in the mutant plants was pale yellow after staining, which was lighter than that in the wild-type plants, indicating that the vitality of viable pollen grains in the mutant plants was lower than that in the wild-type plants. The knockout of the *Ntms1* gene resulted in a significant reduction in the number of viable pollen grains and pollen viability.

**Table 2:** Variance analysis table for pollen grains staining

Lines	Mean (a)	Significant level (5%)	Extremely significant level (1%)
WT	56.6		**
C129	10.8	*	**
C60	9.8	*	**
B23	9.4	*	**
B34	9.4	*	**
B21	7.6	*	**
C125	6.4	*	**

Note: All data are the average of three biological repetitions, a “*” sign represents *P* < 0.05, with a significant difference; a “**” sign represents *P* < 0.01, with a highly significant difference.

#### 3.3.5 A significant decrease in self-pollinated mutants

Self-pollination is the most direct indicator of fertility. The results of the effect of self-pollination on each parameter (Table 3) showed that the yield rate, seed number per fruit, and relative seed setting rate of the mutant plants were significantly lower than those of the wild-type plants; the relative seed setting rate was only 22.03–42.90% of that of the wild-type control. A possible reason is the smaller number of fertile pollen grains in the mutant plants, indicating that the knockout of the *Ntms1* gene led to a decrease in the number of fertile pollen grains and a significant reduction in the self-pollination rate.

**Table 3:** Statistics of self-fertilization of mutants

Lines	Fruit rate (%)	Number of seeds per fruit (seeds)	Relative seed setting rate (%)
WT	100	3074.30	100.00
C129	80**	1319.00**	42.90**
C60	80**	1170.20**	38.06**
B23	70**	1015.50**	33.03**
B34	70**	885.50**	28.80**
B21	70**	770.60**	25.07**
C125	60**	677.30**	22.03**

Note: All data are the average of three biological repetitions, a “*” sign represents *P* < 0.05, with a significant difference; a “**” sign represents *P* < 0.01, with a highly significant difference.

## 4 Discussion

Bioinformatic methods were used to analyze genomic sequences and explore genetic information, predict biological functions of genes, and improve the efficiency of the identification of gene functions [21]. The homologous gene *Zmms1* in maize was cloned by the homologous cloning of *Osms1* in rice. Bioinformatics analysis showed that both *Zmms1* and *Osms1* were very similar and members of the PHD domain family. It is speculated that *Zmms1* may have the same function as *Osms1* in controlling fertility [5], and stable male-sterile lines in maize were obtained by the CRISPR/Cas9 system [6]. In this study, the open reading frame (ORF) of the *Cams1* gene in pepper and the *Ntms1* gene in tobacco were identified using the ORF from the NCBI database. Due to similar physicochemical properties such as the protein isoelectric point, protein domain, and extinction and instability coefficients, it was predicted that the *Ntms1* gene might have the same function as the *Cams1* gene in controlling fertility. The results of the CRISPR/Cas9 gene-editing technology showed that the bioinformatics analysis was a reliable method for predicting the function of homologous genes and can also improve the efficiency of the identification of gene functions.

The *MS1* gene was proven to be a key regulator of pollen development in major crops such as rice, maize, rape, wheat, and pepper. Studies have found that the *MS1* gene encodes a PHD-containing nuclear protein [2]. PHD was closely related to pollen wall composition and tapetum development and did not affect other floral organs but caused pollen abortion [3]. The knockout of the *MS1* gene resulted in atrophy of the anther in rice, lowering the number of anthers compared to the stigma, thus preventing self-pollination [22]. The *MS1* gene knockout also caused the pollen number and pollen viability in *Cucurbita pepo* to decrease [23]. Additionally, due to the knockout, anthers were found to be missing, and pollen sac cells in maize flowers remained undifferentiated [24]. Moreover, the anthers of rice were white and short, with no mature pollen grains, and the seed setting rate decreased significantly [25]. The MDA content in cotton increased significantly, causing abnormal metabolism and accumulation and peroxidation of membrane lipids, thus resulting in pollen abortion [20]. In this study, the *Ntms1* gene of the tobacco variety K326 was knocked out by the CRISPR/Cas9 gene-editing technology. It was found that the expression of the *Ntms1* gene in mutant plants was significantly reduced. The filaments of the floral organs were shortened, and anthers had shrunk; the development of the pollen wall and tapetum were abnormal, and the chamber was severely squeezed. Moreover, the number of viable pollen grains was significantly reduced, and there was also a reduction in the pollen viability; the self-fertility rate was extremely low, and some mutant flowers were even self-infertile, indicating that the *Ntms1* gene regulated fertility in tobacco.

The CRISPR/Cas9 gene-editing technology was used to identify the gene functions. If the gene affects quantitative traits, the T_1_ generation strain should be used, and the test should be performed according to the unique difference principle to obtain accurate identification results. In this study, since the fertility of tobacco plants was the research interest and self-sterility in pollen could not be achieved, the obtained T_1_ generation strain eliminated sterile pollen by natural selection, which no longer provided a strict random population sample. Therefore, the direct identification of sterile mutant traits by using mutant strains of the T_0_ generation was highly reliable in this study.

## 5 Conclusion

In this study, the *Ntms1* gene was cloned from tobacco, and bioinformatics analysis was performed to determine whether the *Ntms1* gene in tobacco and the sterile gene *Cams1* in pepper control fertility similarly. Sterile mutant plants were obtained by the CRISPR/Cas9 gene-editing technology. It was found that the expression level of the mutant *Ntms1* gene was significantly reduced. The filaments of the floral organ were shortened, the anthers were shrunk, and the development of the pollen wall and the tapetum was abnormal. Furthermore, the chamber was severely squeezed, there was a significant reduction in the number of viable pollen grains, and the pollen vitality was also reduced. The self-fertilization rate was extremely low, and some plants were even self-infertile, proving that the *Ntms1* gene regulates fertility in tobacco plants. The results of this research have immense scientific significance and practical value as they provide a basis for further research on the mechanism of the induction and development of new sources of male sterility in tobacco.

## Appendix: Construction of CRISPR/cas9 vector

(1) Select the target.
(2) Construction of intermediate vector gRNA. The gRNA fragments are obtained by the PCR reaction, and the reaction system and procedures are listed in Tables A1 and A2.
(3) Enzyme digestion connection, connection system, and reaction procedure are listed in Tables A3 and A4.
(4) The recombinant plasmid was transformed.
(5) The plasmid was extracted.
(6) Sequencing.
(7) The ligation system and reaction procedure are listed in Tables A5 and A6, respectively.
(8) The system of transformation and bacteria detection is listed in Tables A7 and A8.
(9) Pick out the correct clone, extract the plasmid and send it to the company for sequencing.

**Table A1:** gRNA reaction system

Composition of reaction solution	Reaction liquid capacity (µL)
Forward primer	5
Reverse primer	5
ddH_2_O	40
Total volume	50

**Table A2:** Reaction procedure

Step	Temperature (°C)	Time (min)
Denaturation	95	10
Annealing	55	10
Cooling	14	5

**Table A3:** Enzyme digestion system

Composition of reaction solution	Reaction liquid capacity (µL)
gRNA fragment	2
Empty carrier1 (pBWA(V)HS-zmpl)	1.5
ECO31I	0.5
T4-ligase	0.5
T4-buffer	1
ddH_2_O	4.5
Total volume	10

**Table A4:** Reaction procedure

Step	Temperature (°C)	Time (min)
Denaturation	95	10
Annealing	55	10
Cooling	14	5

**Table A5:** Double enzyme digestion system

Composition of reaction solution	Reaction liquid capacity (µL)
Target 1 (plasmid)	1
Target 2 (plasmid)	1.5
LguI	0.5
T4-ligase	0.5
T4-buffer	1
ddH_2_0	5.5
Total volume	10

**Table A6:** Reaction procedure

Step	Temperature (°C)	Time (min)
Denaturation	95	10
Annealing	55	10
Cooling	14	5

**Table A7:** Bacterial test system

Composition of reaction solution	Reaction liquid capacity (µL)
2× Mix	10
M13F-chen (Forward detection primer)	1
zmpl-chen (Reverse detection primer)	1
ddH_2_O	8
Total volume	20

**Table A8:** Reaction procedure

Step	Temperature (°C)	Time (min)
Denaturation	95	10
Annealing	55	10
Cooling	14	5
